# Supplementary material for: Combined Earth observations reveal the sequence of conditions leading to a large algal bloom in Lake Geneva
Source: Commun Earth Environ. 2024 May 1;5(1):229. doi: 10.1038/s43247-024-01351-5 (PMC11062928; doi:10.1038/s43247-024-01351-5)
Supplement: Supplementary file 2 — Supplementary Information [file 43247_2024_1351_MOESM2_ESM.pdf]

Combined Earth observations reveal the sequence of conditions leading to a large algal bloom in Lake Geneva

Supplementary Information

Abolfazl Irani Rahaghi[1,2,], Daniel Odermatt[1,2], Orlane Anneville[3], Oscar Sepúlveda Steiner[4,5], Rafael Sebastian Reiss[6], Marina Amadori[7], Marco Toffolon[8], Stéphan Jacquet[3], Tristan Harmel[9], Mortimer Werther[1], Frédéric Soulignac[10], Etienne Dambrine[3], Didier Jézéquel[3,11], Christine Hatté[12,13], Viet Tran-Khac[3], Serena Rasconi[3], Frédéric Rimet[3], Damien Bouffard[4]*

[1] Eawag, Swiss Federal Institute of Aquatic Science & Technology, Surface Waters – Research and Management, 8600 Duebendorf, Switzerland
[2] Department of Geography, University of Zurich, 8057 Zurich, Switzerland
[3] Université Savoie Mont Blanc, INRAE, UMR CARRTEL, 74200 Thonon les Bains, France
[4] Eawag, Swiss Federal Institute of Aquatic Science & Technology, Surface Waters – Research and Management, 6047 Kastanienbaum, Switzerland
[5] Department of Civil & Environmental Engineering, University of California - Davis, Davis, CA, USA
[6] Ecological Engineering Laboratory (ECOL), Institute of Environmental Engineering (IIE), Faculty of Architecture, Civil and Environmental Engineering (ENAC), Ecole Polytechnique Fédérale de Lausanne (EPFL), 1015 Lausanne, Switzerland
[7] Institute for Electromagnetic Sensing of the Environment (IREA), National Research Council of Italy (CNR), 20133 Milan, Italy
[8] Department of Civil, Environmental and Mechanical Engineering, University of Trento, 28122 Trento, Italy
[9] Earth Observation Unit, Magellium, Toulouse, France
[10] Commission Internationale pour la Protection des Eaux du Léman (CIPEL), Nyon, Switzerland
[11] Université Paris Cité, Institut de Physique du Globe de Paris, CNRS, 75005 Paris, France
[12] Laboratoire des Sciences du Climat et de l'Environnement, CEA, CNRS, UVSQ, Université Paris-Saclay, 91191 Gif-sur-Yvette, France
[13] Institute of Physics, Silesian University of Technology, 44-100 Gliwce, Poland

*Corresponding author: abolfazl.irani@eawag.ch

Supplementary Methods - Hydrodynamic model validation and evaluation

The employed 3D hydrodynamic model has been calibrated on a long-term analysis implementing Bayesian inference together with a smoothed particle Markov Chain Monte Carlo method[1]. In the original model evaluation, the model results were compared with one year temperature and current measurements in Lake Geneva. Building on such well-established model setup, here, we demonstrate the model suitability for simulating the temperature and flow-field during the shorter period under investigation, i.e., August 18-September 8, 2021. We rely on in-situ measurements from an acoustic Doppler current profiler (ADCP) and thermistor chain at the *LéXPLORE* station (red square in Figure 1), as well as temperatures at 1 m depth at *Buchillon* station (red star in Figure 1). The measurement frequencies of those datasets are 10 min, 10 min, and 1 hr, respectively. Statistical measures, such as distribution coherency, root mean square error (RMSE), Pearson correlation coefficient (R-value), and bias are employed to evaluate the model's performance. The in-situ measurements were resampled at the model output timestamps to calculate those metrics.

Horizontal velocity components measured between 11-30 m depth by an ADCP at the *LéXPLORE* station (https://www.datalakes-eawag.ch/datadetail/599) were used for further analysis. Above 11 m there are no reliable velocity measurements. This depth range allows us to better assess the model performance in reproducing the surface layer currents as the main contributor to the bloom advection (presented in Figure 2A-C and discussed in the text). Comparison of the temporal variation of velocity magnitude (Figure S1) shows that the model results failed to follow the observations' peaks until around August 17. The temporal evolution of estimated daily RMSE reaches an almost stabilized asymptotic point on this date (Figure S1B), which is consistent with the model's long term RMSE performance of 0.033 [m s^{-1}][1]. This period, i.e., July 26 to Aug 17, can be recognized as the model spin up time. The distribution of obtained velocity magnitude and direction from numerical simulation during the rest of the modeling period, i.e., August 18 to September 8, is strongly correlated with the in-situ measurements (Figure S2). The calculated correlation values are 0.95 and 0.96 for velocity magnitude and velocity direction, respectively.

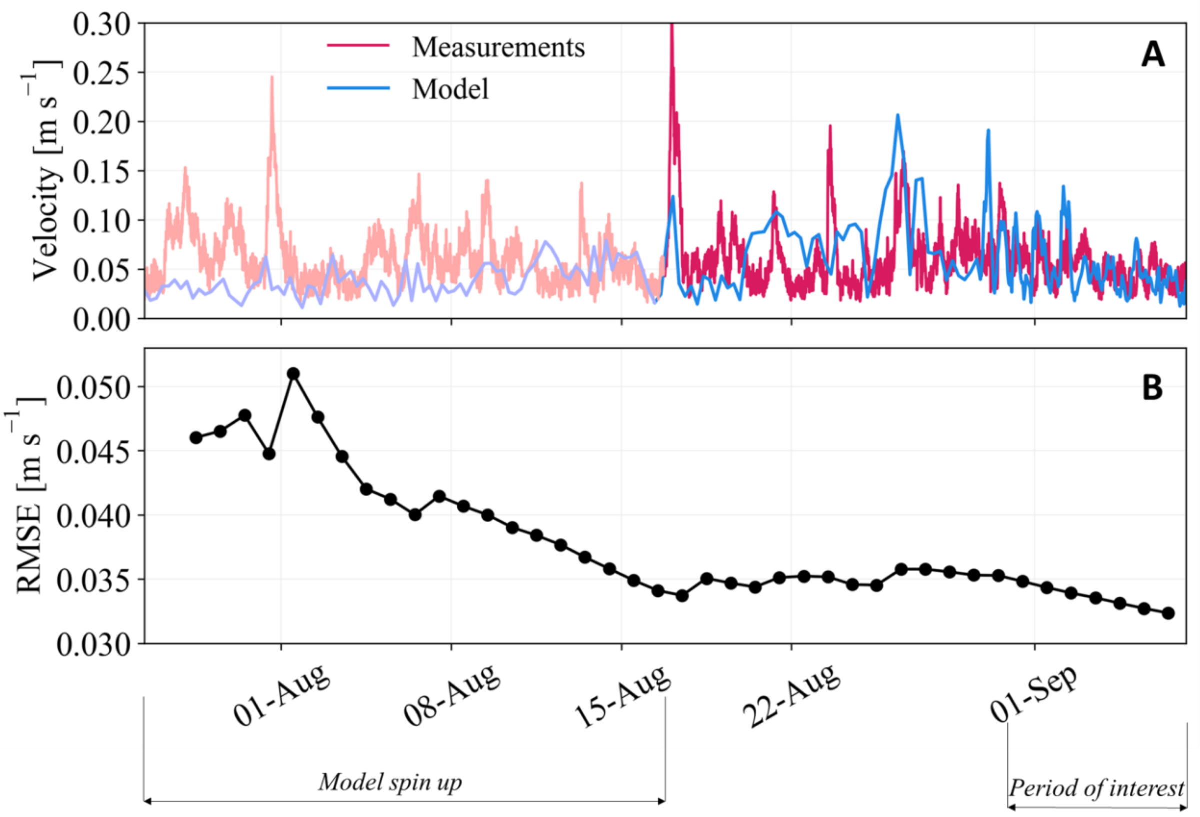

Supplementary Figure 1. (**A**) Comparison of measured (red lines) and modeled (blue lines) velocities averaged between 11 to 30 m after model initialization on July 26, 2021, and (**B**) evolution of daily root mean square error (RMSE) of modeled velocities with respect to observations. The measurements are from an acoustic Doppler current profiler (ADCP) deployed at the *LéXPLORE* platform. The lack of strong correlation between model outputs and measurements during the first 3 weeks has been indicated as "*Model spin up*" time. "*Period of interest*" shows the duration of 3D numerical model results used for analyses and particle tracking, i.e., August 31 to September 6.

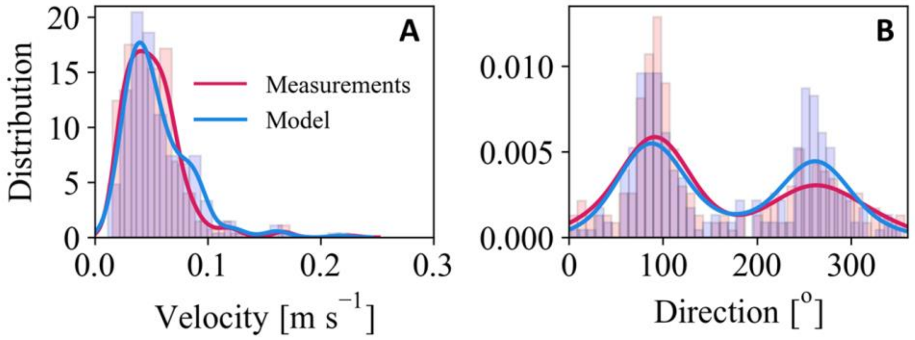

Supplementary Figure 2. Comparison of the distribution of velocity magnitude (**A**) and direction (**B**) between measurements (red bars and lines) and model results (blue bars and lines) during August 18 and September 8, 2021. The lines indicate estimated kernel density (as a proxy for probability density function).

The evolution of temperatures and thermal structures during August 18 to September 8 at the *LéXPLORE* station were also compared between in situ measurements (https://www.datalakes-eawag.ch/datadetail/448) and numerical model results. In general, model results follow the observed temperature variation and stratification (Figure S3) with some discrepancies, e.g., around August 24-25.

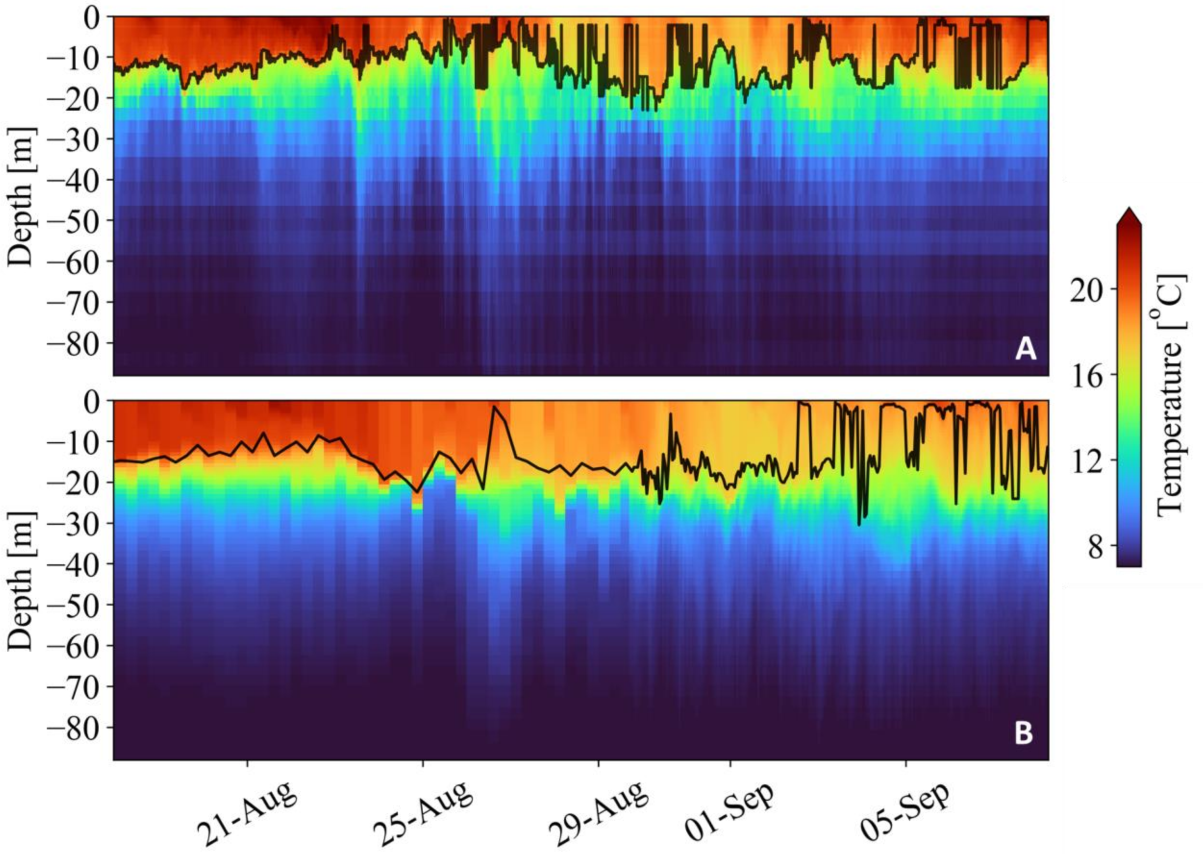

Supplementary Figure 3. Evolution of temperature profile between August 18 and September 8, 2021 at the *LéXPLORE* station (Figure 1) using measured (**A**) and modeled (**B**) temperatures. The solid back lines indicate the estimated thermocline depth.

For a better quantitative assessment, the time series of estimated mean epilimnion temperature ($T_{epilimnion}$), mean hypolimnion temperature ($T_{hypolimnion}$), and Schmidt stability ($S_{Schmidt}$) were compared (Figure S4). Those values were derived following Read et al[2]. The Schmidt stability represents the energy necessary to fully mix the lake[3,4]. It varies in time depending on the lake density stratification and on the hypsometric curve:

$$S_{Schmidt} = \frac{g}{A_0}\int_0^{Z_L} (z - z_v)\rho_z A_z dz \tag{S1}$$

where *g* is the gravitational acceleration, A_0 is the surface extension, A_z is the area associated with depth $z = z_v$, and z_L is the maximum depth of the lake. The results demonstrate a relatively good agreement between model and observations using different performance metrics (Table S1) with the best correlation coefficient of 0.93 for the mean hypolimnion temperature, and the worst correlation coefficient of 0.41 for the Schmidt stability. The model results show an overestimation of Schmidt stability, which is a sign of stronger stratification by the model. However, the performance metrics during the "*period of interest*" (given in parentheses in Table S1) are better for $T_{hypolimnion}$, and $S_{Schmidt}$, and slightly worse for $T_{epilimnion}$.

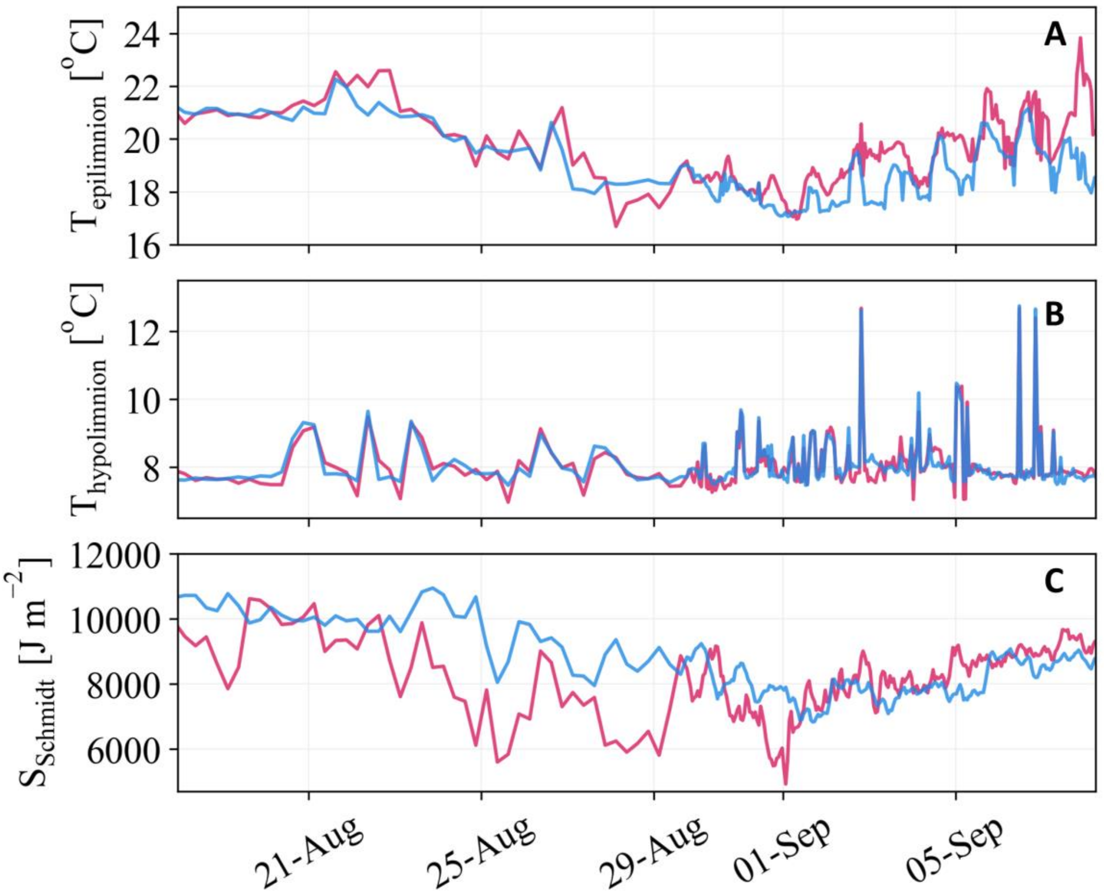

Supplementary Figure 4. Comparison of measured (red lines) and modeled (blue lines) mean epilimnion temperature (**A**), mean hypolimnion temperature (**B**), and Schmidt stability (**C**) at the *LéXPLORE* station (Figure 1) between August 18 and September 8, 2021. The model's performance metrics are given in Table S1.

Supplementary Table 1. Selected performance metrics of the 3D hydrodynamic model by comparing the modeled and measured mean epilimnion temperature ($T_{epilimnion}$), mean hypolimnion temperature ($T_{hypolimnion}$), and Schmidt stability ($S_{Schmidt}$) at the *LéXPLORE* station (Figure 1) between August 18 and September 8, 2021 (Figure S4). The values in parentheses indicate the performance metrics for the "*Period of interest*" (Figure S1).

	RMSE	R-value	Bias
$T_{epilimnion}$ [^{o}C]	1.44 (1.61)	0.68 (0.63)	-0.93 (-1.2)
$T_{hypolimnion}$ [^{o}C]	0.23 (0.21)	0.93 (0.95)	0.02 (0.0)
$S_{Schmidt}$ [J m^{-2}]	1082 (633)	0.41 (0.62)	127 (-198)

The measured water temperatures at 1m depth at the *Buchillon* station (https://www.datalakes-eawag.ch/datadetail/597) were also compared with numerical models after the model spun up (Figure S5). The model accurately follows the general trend and diurnal variation of the observation, albeit with a bias of -0.52 °C.

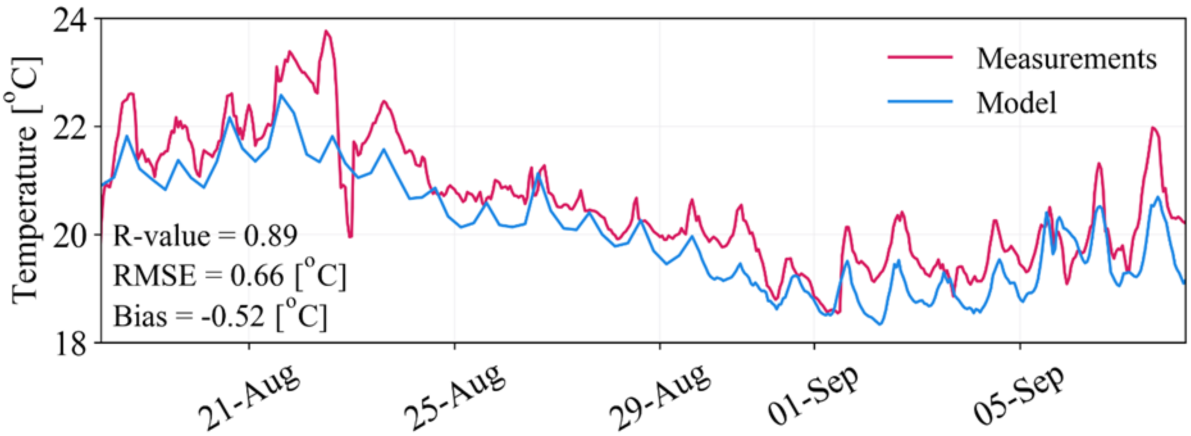

Supplementary Figure 5. Comparison of measured (red lines) and modeled (blue lines) temperatures at 1 m depth at the *Buchillon* station (Figure 1) between August 18 and September 8, 2021. The model's performance metrics including correlation coefficient (R-value), root mean square error (RMSE), and bias are given.

Supplementary Note 1- The observed circulation structure

During the relaxation phase following the wind event, the resulting circulation took a three-gyre structure (Figure 2B), which contributed significantly to the transport and dispersion of phytoplankton bloom from littoral to pelagic zone. Particle tracking (Figure 2A) revealed that the advective transport of the central pattern was faster compared to the western mushroom-like pattern, resulting in its dilution towards the northern shore. This is because the anticyclonic basin-scale circulation in the center is stronger than the one in the west (denoted by larger arrows in Figure 2B). The eastern basin-scale cyclonic gyre, however, creates a pelagic upwelling region, and therefore a higher resistance to the lateral surface water infiltration. Such feature can explain the low concentration quasi-circular pattern shaped with the forward particle tracking in the region of this cyclonic gyre (Figure 2A). These particle tracking results are in line with the satellite observation indicating an eastern basin-scale circular pattern with elongated filaments along its edge (Figure 1). Our backward tracking results (Figure S11C) showed that the particles in this gyre have an origin most likely from the main tributary of the lake, i.e., *Rhône* (in) in Figure 1, entering this region more than five days before the peak of the bloom.

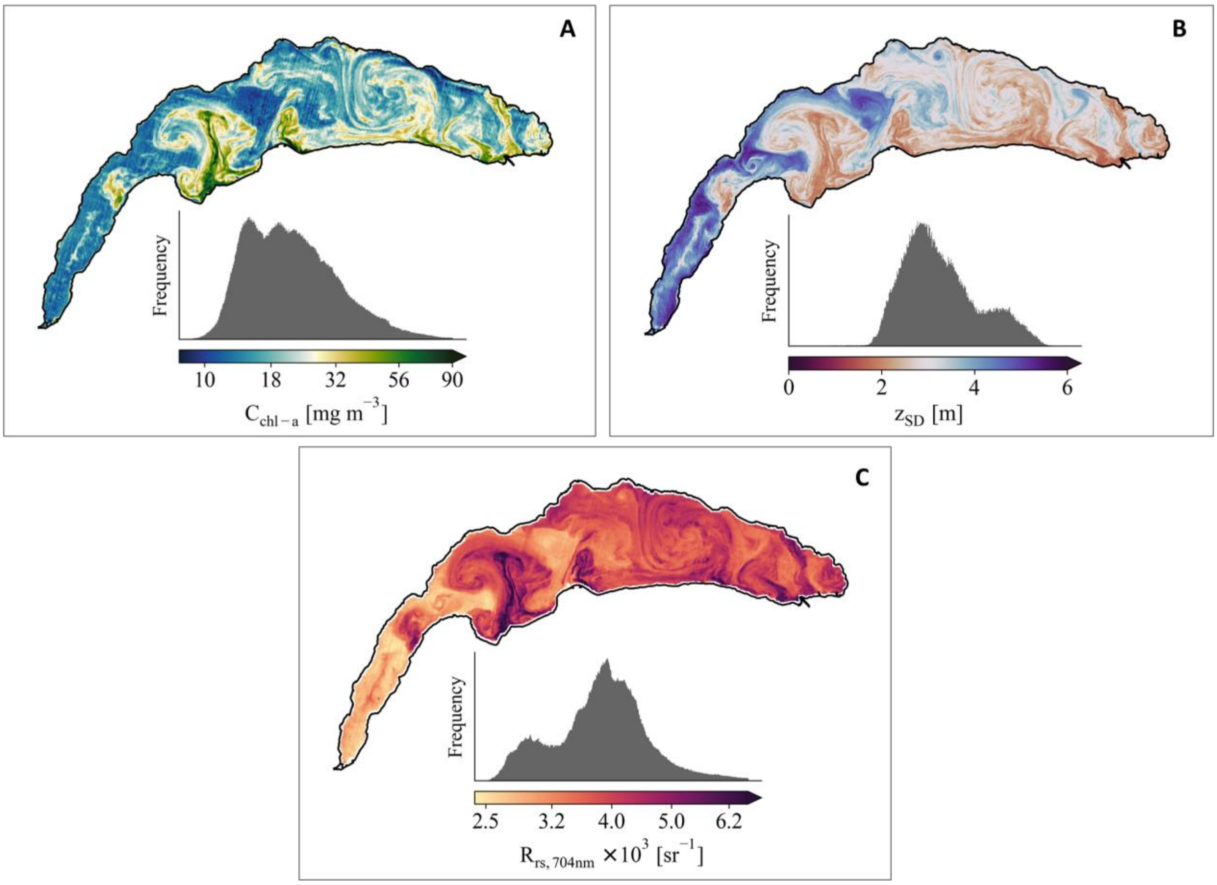

Supplementary Figure 6. Maps of **(A)** estimated chlorophyll-a concentrations ($C_{chl\text{-}a}$), **(B)** estimated Secchi depth (z_{SD}), and **(C)** remote sensing reflectance at near-infrared 704 nm ($R_{rs,704nm}$) obtained from Sentinel-2 MSI over Lake Geneva on September 6, 2021. The reflectance values at three red and NIR wavelengths (665, 704, and 754 nm) were used to estimate $C_{chl\text{-}a}$[5,6]. The RGB-QAA algorithm[7] was used to obtain z_{SD}. The inset plots show the histogram of the spatial variations. Note that the color bars in **A** and **C** are in logarithmic scale.

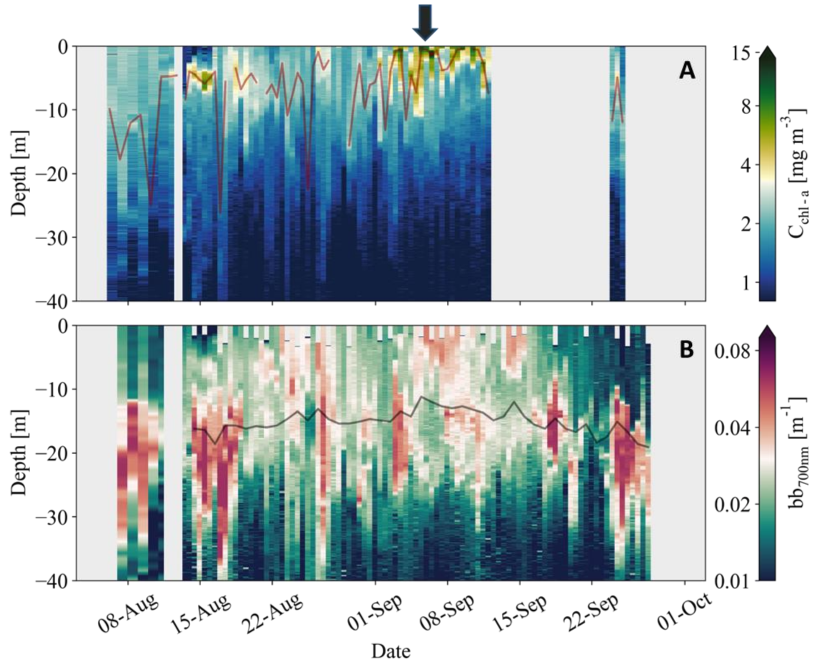

Supplementary Figure 7. Depth profiles of **(A)** chl-a concentration (C_{chl-a}), and **(B)** backscattering coefficient at 700 nm from automated profiler measurements at the *LéXPLORE* platform (red square in Figure 1). The solid red line in **A** indicates the location of estimated subsurface C_{chl-a} maxima. The solid black line in **B** shows the location of estimated euphotic depth (for details, cf. Methods section), respectively. The euphotic depth during the peak of the bloom (September 5-6 indicated with the black arrow in **A**) was ~11.2 m, which is 5-8 m less than before (mid-August) and after (end of September) the bloom.

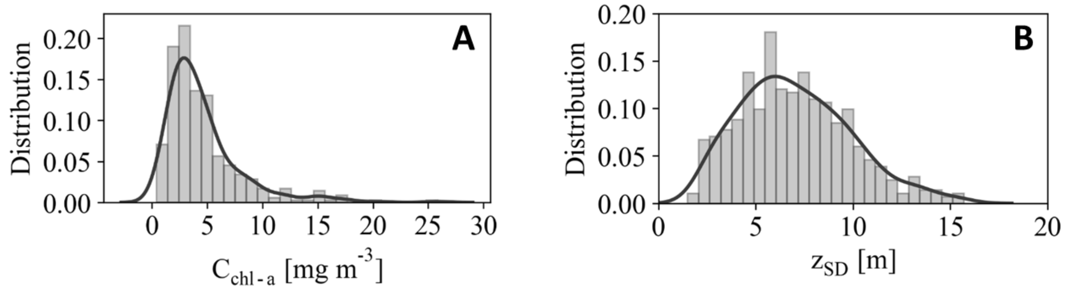

Supplementary Figure 8. Distribution of long-term **(A)** chl-a concentration (C_{chl-a}), and **(B)** Secchi depth (Z_{SD}) measurements at SHL2 (red triangle in Figure 1). The distributions are based on biweekly to monthly measurements during the period 2002-2019.

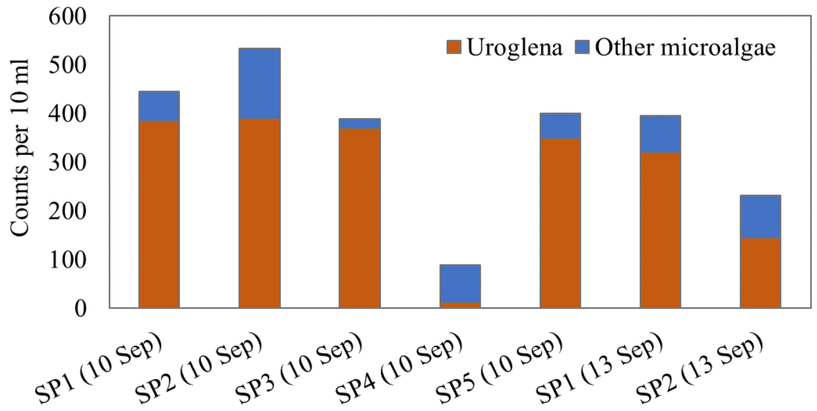

Supplementary Figure 9. Biovolume analysis of water samples from Lake Geneva on 10 and 13 September 2021 at five locations in the lake, i.e., SP1 to SP5 in Figure 1.

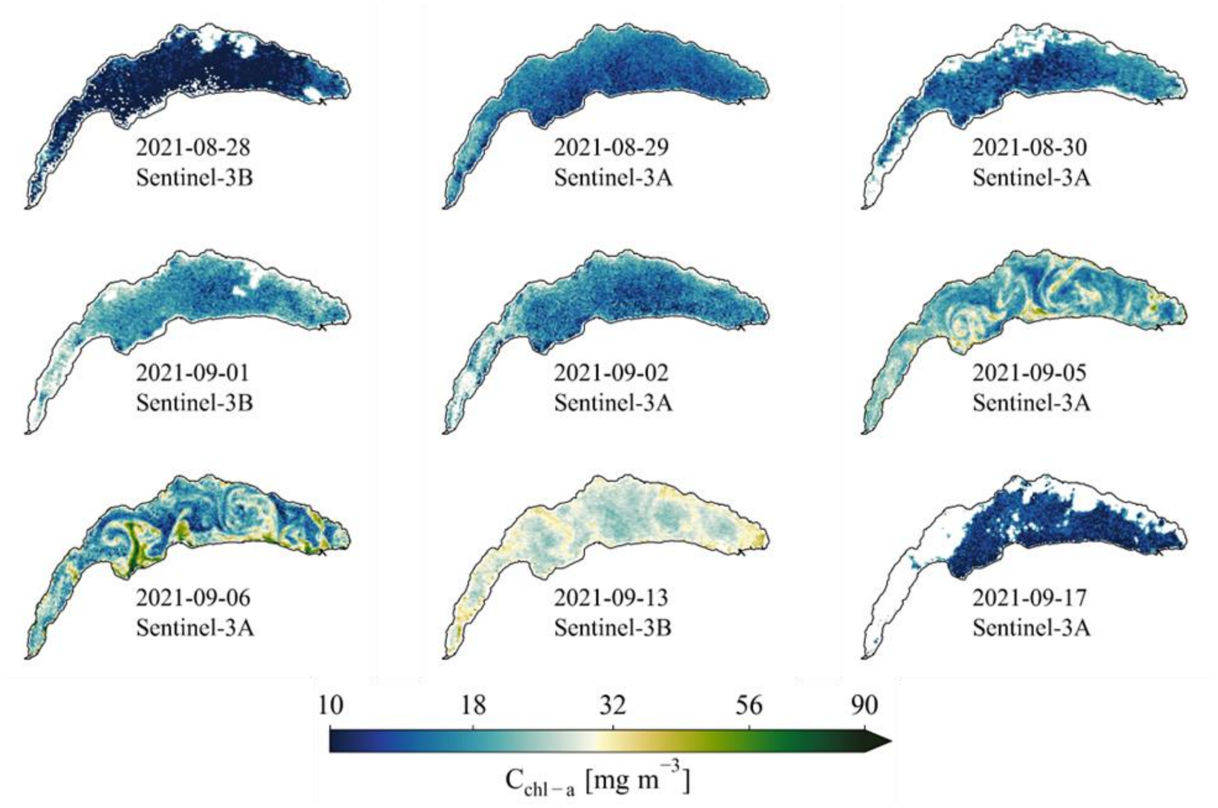

Supplementary Figure 10. Sentinel-3 OLCI satellite images of Lake Geneva between August 21 and September 17, 2021. The maps show the estimated chlorophyll-a concentrations (C_{chl-a}) from remote sensing reflectance products at three red and NIR wavelengths (665, 709, and 754 nm)[5,8].

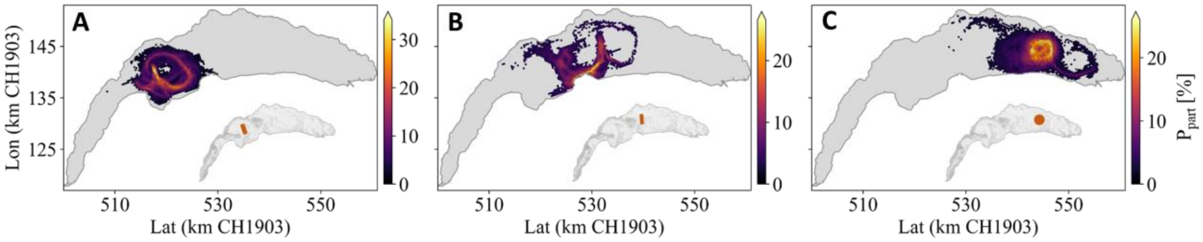

Supplementary Figure 11. Backward particle tracking results for particles seeding in: **(A)** western, **(B)** central, and **(C)** eastern parts of the main basin. The orange areas in the inset plots indicate the seeding points. 200000 particles were instantaneously released on September 6, 2021 at 12:30 in the near-surface layer (0-5 m) at those seeding points, and were tracked backward until 31 August 31, 2021 at 02:30.

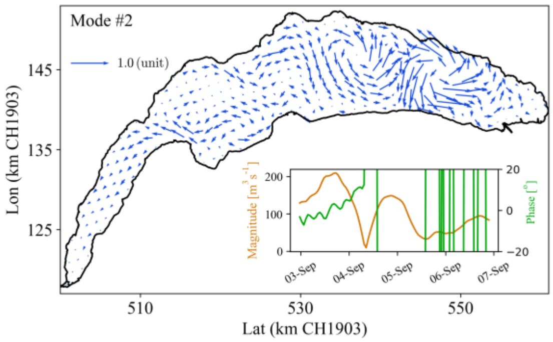

Supplementary Figure 12. Second EOF mode of velocity flux in the top 20 m of the water column. The inset shows the temporal variation of magnitude and phase of the second EOF mode. The key for the spatial quiver plot is given on the top left.

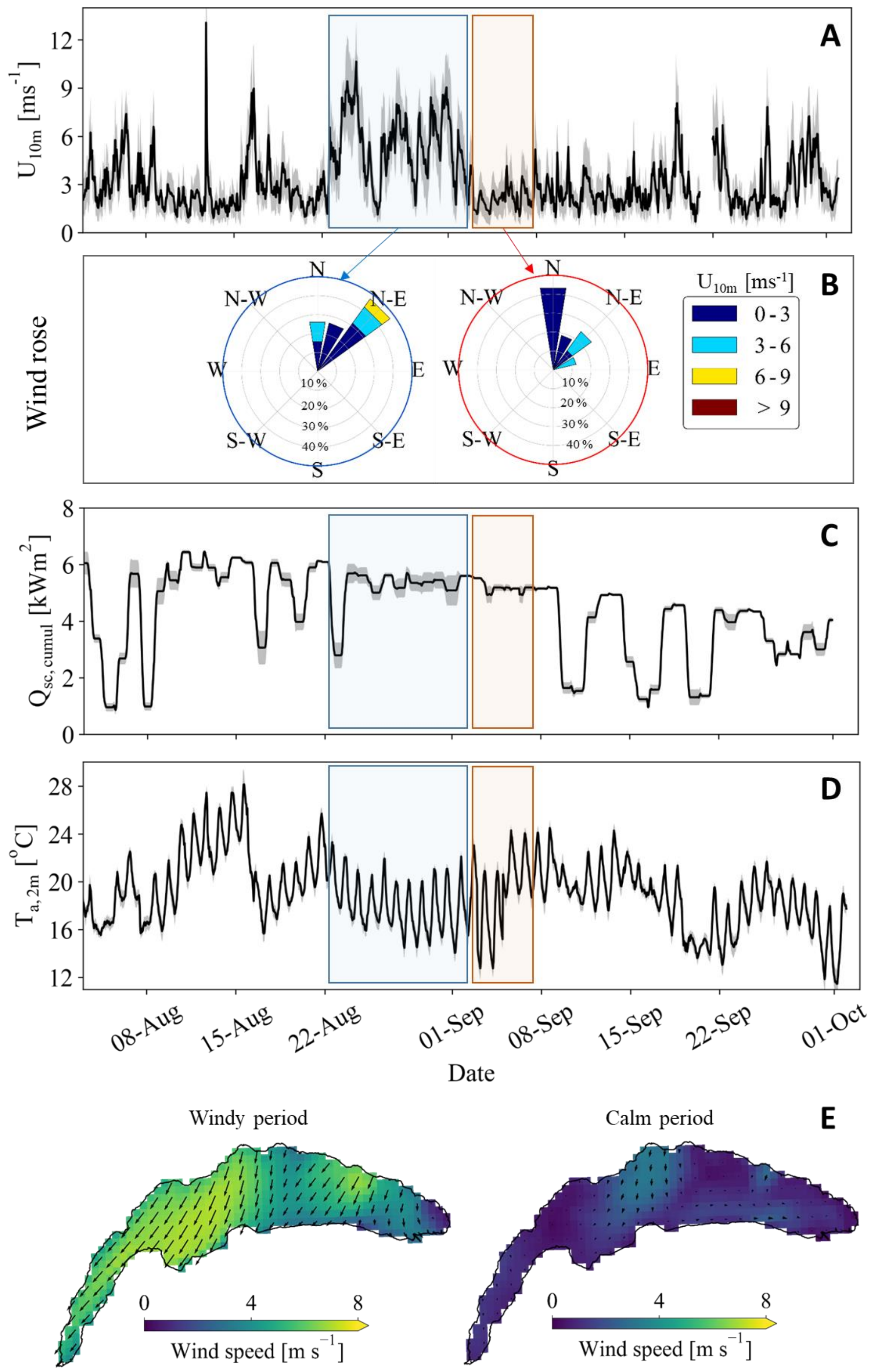

Supplementary Figure 13. Lake-wide averaged meteorological parameters based on hourly reanalysis data from the COSMO numerical weather model: **(A)** wind speed, **(B)** wind rose diagram for two selected periods in **A**, **(C)** global radiation converted to cumulative daily values, and **(D)** air temperature. Similar to in-situ measurements (Figure 3) at the *LéXPLORE* platform (red square in Figure 1), the data show a windy cold period (blue shaded rectangle) followed by a calm warm period (red shaded rectangle) prior to the peak of the bloom. The mean and standard deviation of lake-wide data are indicated with the solid black line and grey shaded area, respectively. The mean spatial wind patterns indicate the prevailing northeasterly winds during the windy period (left panel in **E**) and weak wind over the entire lake during the calm period (right panel in **E**).

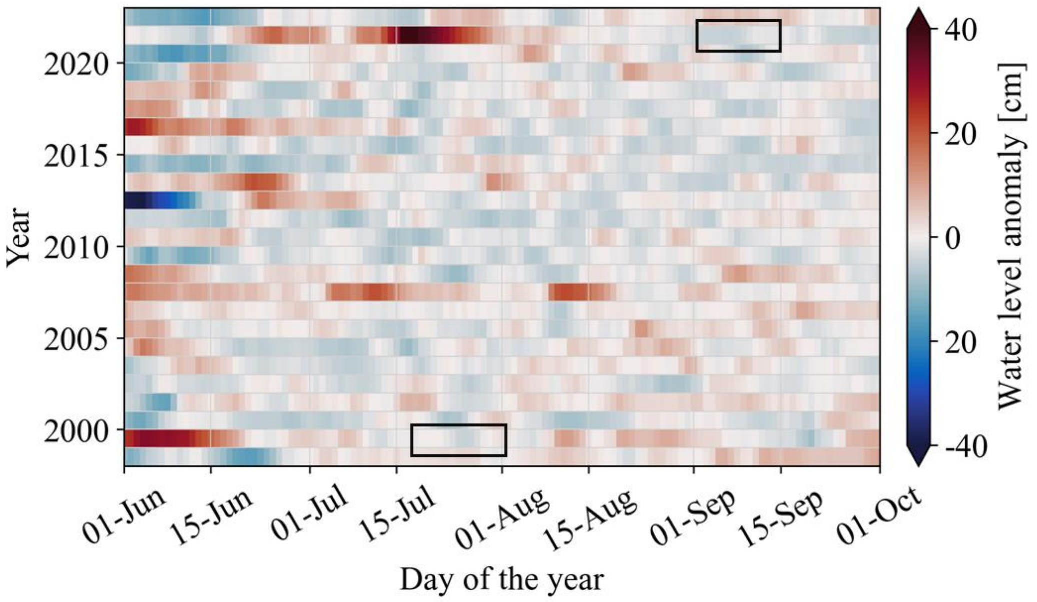

Supplementary Figure 14. Lake water level anomaly variation between 1998-2022. The black rectangles in 1999 and 2021 indicate two occasions when *Uroglena Sp.* algae were reported in Lake Geneva a few weeks after the extreme water level increase.

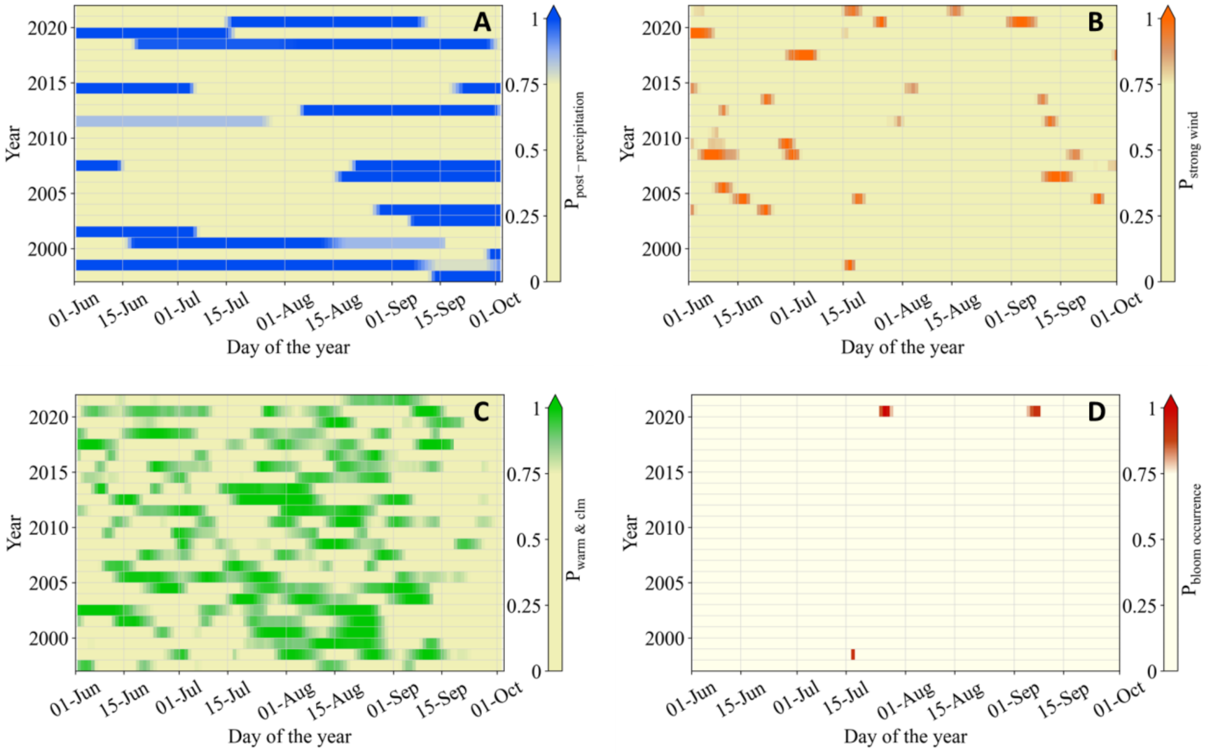

Supplementary Figure 15. The incidents when favorable meteorological conditions for bloom occurrence were satisfied in Lake Geneva during 1998-2022: **(A)** extreme precipitation event a few weeks before (post-precipitation), **(B)** strong wind event a few days before (potential upwelling), **(C)** warm and calm condition for a few days after, and **(D)** calculated probability of bloom occurrence. An arbitrary threshold of > 0.75 was selected in all colormaps.

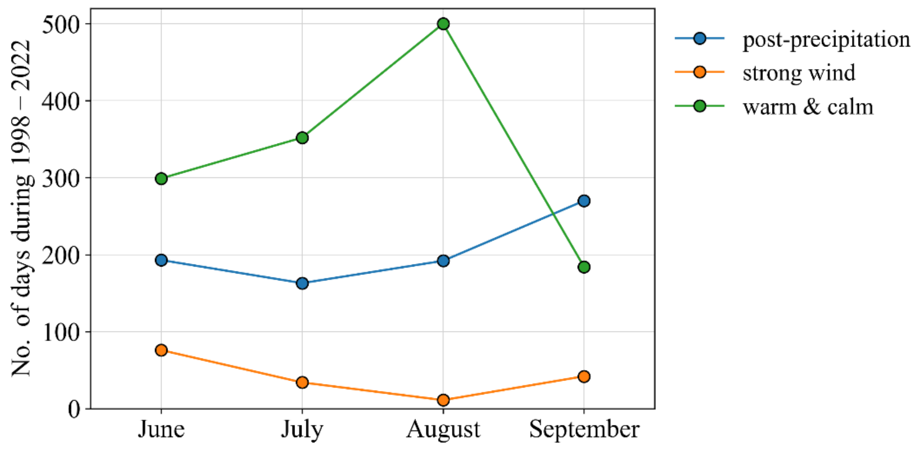

Supplementary Figure 16. The number of monthly events for each of the meteorological forcing conditions required for bloom occurrence during the period 1998-2022.

Supplementary Table 2. Summary of ^{14}C analysis during different phases of the bloom and at different locations in Lake Geneva. The high $F^{14}C$ of the bloom at Sechex littoral compared to bulk littoral soils and water extracts may be explained by the release of bomb-labeled carbon compounds stored[9–11] in subsoil layers such as reworked banks. However, the $F^{14}C$ of the bloom within INRAE harbor, locally protected from bank erosion by large stone embankments was low. The samples taken in the pelagic zone show that the bloom was lately fed by dissolved inorganic carbon[12,13], possibly from water layers lifted by the upwelling and/or from the CO_2 released after calcite bioprecipitation, which occurs commonly in summer when gross primary production peaks[14].

Phases	Sampling date	Site	Nature	OC (mg L^{-1})	C/N	$\delta^{13}C$ [*]	^{14}C activity				
							chemistry #	measurement #	No. of measured aliquots	$F^{14}C$	± [**]
Pre-bloom		Thonon littoral surface	Soil total OM				GifA22239	ECHo-5044	2	1.007	0.005
			Extracted DOC				GifA23162		1	1.038	0.001
		Thonon bank subsurface	Eroded soil total OM				GifA22240	ECHo-5045	2	1.027	0.004
			Extracted DOC				GifA23163		1	1.077	0.001
		Sechex beach surface	Eroded soil total OM				GifA22241	ECHo-5046	2	1.053	0.006
			Extracted DOC				GifA23164		1	1.038	0.001
		Redon river littoral	Sediment (total OM)				GifA22242	ECHo-4959	1	0.929	0.002
Bloom	10/09/2021	Séchex littoral	Surface water POC	0.55	6.7	-25.1	GifA21579	ECHo-4731	2	1.225	0.023
		Séchex pelagic		0.18	6.4	-26.6	GifA21582	ECHo-4734	2	0.809	0.021
		Inside INRA harbor		0.82	6.2	-25.4	GifA21577	ECHo-4729	2	0.876	0.006
		Lausanne Pully littoral		0.30	6.3	-25.5	GifA21581	ECHo-4733	3	0.847	0.009
	13/09/2021	Thonon pelagic		0.72	7.3	-24.4	GifA21580	ECHo-4732	2	0.884	0.006
	15/09/2021	Centre lake SHL2	Lake water POC (-5m)	0.19	8.8	-25.6	GifA21578	ECHo-4730	2	0.853	0.006
		Centre lake SHL2	Daphnia (0-50m)				GifA21576	ECHo-4721	1	0.815	0.009
Post-bloom	19/10/2021	Centre lake SHL2	Surface water POC	0.08		-28.8	GifA21583	ECHo-4735	2	0.871	0.016
		Centre lake SHL2	Daphnia (0-50m)				GifA21575	ECHo-4707	3	0.847	0.006
	20/10/2021	Centre lake SHL2	DIC (0-5m)				GifA21573	SacA-67655	1	0.860	0.003
		Centre lake SHL2	DIC (100m)				GifA21574	SacA-67654	1	0.852	0.003
		Centre lake SHL2	DIC (275-309 m)				GifA21572	SacA-67653	1	0.851	0.003

[*] measured by IRMS, typical error of 0.1‰

[**] = max between the standard deviation of individual measurements and typical individual measurement error)

Supplementary References

1. Safin, A. *et al.* A Bayesian data assimilation framework for lake 3D hydrodynamic models with a physics-preserving particle filtering method using SPUX-MITgcm v1. *Geosci. Model Dev.* **15**, 7715–7730 (2022).
2. Read, J. S. *et al.* Derivation of lake mixing and stratification indices from high-resolution lake buoy data. *Environ. Model. Softw.* **26**, 1325–1336 (2011).
3. Schmidt, W. Über die Temperatur- und Stabilitätsverhältnisse von Seen. *Geografiska. Annaler.* **10**, 145 (1928).
4. Idso, S. B. On the concept of lake stability. *Limnol. Oceanogr.* **18**, 681–683 (1973).
5. Ogashawara, I. *et al.* The use of Sentinel-2 for chlorophyll-a spatial dynamics assessment: A comparative study on different lakes in Northern Germany. *Remote Sens.* **13**, 1542 (2021).
6. Dall'Olmo, G. & Gitelson, A. A. Effect of bio-optical parameter variability on the remote estimation of chlorophyll-a concentration in turbid productive waters: experimental results. *Appl. Opt.* **44**, 412 (2005).
7. Pitarch, J. & Vanhellemont, Q. The QAA-RGB: A universal three-band absorption and backscattering retrieval algorithm for high resolution satellite sensors. Development and implementation in ACOLITE. *Remote Sens. Environ.* **265**, 112667 (2021).
8. Gilerson, A. A. *et al.* Algorithms for remote estimation of chlorophyll-a in coastal and inland waters using red and near infrared bands. *Opt. Express* **18**, 24109–24125 (2010).
9. Trumbore, S. E. Comparison of carbon dynamics in tropical and temperate soils using radiocarbon measurements. *Global Biogeochem. Cycles* **7**, 275–290 (1993).
10. Trumbore, S. Radiocarbon and Soil Carbon Dynamics. *Annu. Rev. Earth Planet. Sci.* **37**, 47–66 (2009).
11. Butman, D., Raymond, P., Oh, N.-H. & Mull, K. Quantity, 14C age and lability of desorbed soil organic carbon in fresh water and seawater. *Org. Geochem.* **38**, 1547–1557 (2007).
12. Aho, K. S., Hosen, J. D., Logozzo, L. A., McGillis, W. R. & Raymond, P. A. Highest rates of gross primary productivity maintained despite CO_2 depletion in a temperate river network. *Limnol. Oceanogr. Letters* **6**, 200–206 (2021).
13. Perolo, P. *et al.* Alkalinity contributes at least a third of annual gross primary production in a deep stratified hardwater lake. *Limnol. Oceanogr. Letters* **8**, 359–367 (2023).
14. Escoffier, N., Perolo, P., Many, G., Pasche, N. T. & Perga, M.-E. Fine-scale dynamics of calcite precipitation in a large hardwater lake. *Sci. Total Environ.* **864**, 160699 (2023).
