# Supplementary material for: Combined Earth observations reveal the sequence of conditions leading to a large algal bloom in Lake Geneva
Source: Commun Earth Environ. 2024 May 1;5(1):229. doi: 10.1038/s43247-024-01351-5 (PMC11062928; doi:10.1038/s43247-024-01351-5)
Supplement: Supplementary file 3 — Description of Additional Supplementary Files [file 43247_2024_1351_MOESM3_ESM.pdf]

Description of Additional Supplementary Files

File name: Supplementary Movie 1

File Description: Tracking the fate of particles released hourly in the top 10 m of water column in the southern shore of Lake Geneva between September 3 to September 6, 2021.

File name: Supplementary Movie 2

File Description: Tracking the origin of particles released on September 6, 2021 at 12:30 in the near-surface layer (0-5 m) at the center of western circulation pattern in Lake Geneva.

File name: Supplementary Movie 3

File Description: Tracking the origin of particles released on September 6, 2021 at 12:30 in the near-surface layer (0-5 m) at the center of central circulation pattern in Lake Geneva.

File name: Supplementary Movie 4

File Description: Tracking the origin of particles released on September 6, 2021 at 12:30 in the near-surface layer (0-5 m) at the center of eastern circulation pattern in Lake Geneva.
