# Supplementary material for: Combined Earth observations reveal the sequence of conditions leading to a large algal bloom in Lake Geneva
Source: Commun Earth Environ. 2024 May 1;5(1):229. doi: 10.1038/s43247-024-01351-5 (PMC11062928; doi:10.1038/s43247-024-01351-5)
Supplement: Supplementary file 8 — Reporting summary [file 43247_2024_1351_MOESM8_ESM.pdf]

nature portfolio

Corresponding author(s): Abolfazl Irani Rahaghi

Last updated by author(s): Feb 25, 2024

Reporting Summary

Nature Portfolio wishes to improve the reproducibility of the work that we publish. This form provides structure for consistency and transparency in reporting. For further information on Nature Portfolio policies, see our Editorial Policies and the Editorial Policy Checklist.

Statistics

For all statistical analyses, confirm that the following items are present in the figure legend, table legend, main text, or Methods section.

n/a	Confirmed	
☐	☒	The exact sample size (*n*) for each experimental group/condition, given as a discrete number and unit of measurement
☐	☒	A statement on whether measurements were taken from distinct samples or whether the same sample was measured repeatedly
☒	☐	The statistical test(s) used AND whether they are one- or two-sided<br>*Only common tests should be described solely by name; describe more complex techniques in the Methods section.*
☒	☐	A description of all covariates tested
☒	☐	A description of any assumptions or corrections, such as tests of normality and adjustment for multiple comparisons
☒	☐	A full description of the statistical parameters including central tendency (e.g. means) or other basic estimates (e.g. regression coefficient) AND variation (e.g. standard deviation) or associated estimates of uncertainty (e.g. confidence intervals)
☒	☐	For null hypothesis testing, the test statistic (e.g. *F*, *t*, *r*) with confidence intervals, effect sizes, degrees of freedom and *P* value noted<br>*Give P values as exact values whenever suitable.*
☒	☐	For Bayesian analysis, information on the choice of priors and Markov chain Monte Carlo settings
☒	☐	For hierarchical and complex designs, identification of the appropriate level for tests and full reporting of outcomes
☒	☐	Estimates of effect sizes (e.g. Cohen's *d*, Pearson's *r*), indicating how they were calculated

Our web collection on statistics for biologists contains articles on many of the points above.

Software and code

Policy information about availability of computer code

Data collection	No software was used.
Data analysis	The main hydrodynamic code is available at a modified MITgcm repository21: https://renkulab.io/projects/artur.safin/DatalakesHydrodynamics. Lagrangian C-tracker particle tracking code is available at https://zenodo.org/record/1034118. Python 3 was used for all data cleaning, processing, and post-processing.

For manuscripts utilizing custom algorithms or software that are central to the research but not yet described in published literature, software must be made available to editors and reviewers. We strongly encourage code deposition in a community repository (e.g. GitHub). See the Nature Portfolio guidelines for submitting code & software for further information.

Data

Policy information about availability of data

All manuscripts must include a data availability statement. This statement should provide the following information, where applicable:

- Accession codes, unique identifiers, or web links for publicly available datasets
- A description of any restrictions on data availability
- For clinical datasets or third party data, please ensure that the statement adheres to our policy

The LéXPLORE and Buchillon datasets are openly available at www.datalakes-eawag.ch. The source data for regenerating the graphs and plots in the manuscript will be available for download at https://doi.org/10.25678/000C9Q upon the acceptance of our manuscript. Long-term systematic water quality measurements at SHL2

were obtained from https://si-ola.inrae.fr/si_lacs/login.jsf. Reanalysis surface forcing COSMO data can be requested from MeteoSwiss (https://www.meteoswiss.admin.ch/). Water level data can be demanded in the "Hydrological Data Service" section of BAFU/FOEN website: https://www.bafu.admin.ch/.

Human research participants

Policy information about studies involving human research participants and Sex and Gender in Research.

Reporting on sex and gender	N/A
Population characteristics	N/A
Recruitment	N/A
Ethics oversight	N/A

Note that full information on the approval of the study protocol must also be provided in the manuscript.

Field-specific reporting

Please select the one below that is the best fit for your research. If you are not sure, read the appropriate sections before making your selection.

☐ Life sciences ☐ Behavioural & social sciences ☒ Ecological, evolutionary & environmental sciences

For a reference copy of the document with all sections, see nature.com/documents/nr-reporting-summary-flat.pdf

Ecological, evolutionary & environmental sciences study design

All studies must disclose on these points even when the disclosure is negative.

Study description	This study examines a recent massive Uroglena bloom in Lake Geneva (Switzerland/France). We show that a certain sequence of meteorological conditions triggered this specific algal bloom event: heavy rainfall promoting excessive organic matter and nutrients loading, followed by wind-induced coastal upwelling, and a prolonged period of warm, calm weather. The combination of satellite remote sensing, in-situ measurements, ad-hoc biogeochemical analyses, and three-dimensional modeling proved invaluable in unraveling the complex dynamics of algal blooms highlighting the significant role of littoral-pelagic connectivities in large low-nutrient lakes.
Research sample	To identify the taxonomy of the algal bloom water samples were taken from Lake Geneva in Five locations and at different depths (close to the surface, the depth of maximum chlorophyll-a concentration, and/or metalimnion) on September 10, 2021 and September 13, 2021. Soil and sediment were also sampled with a spade and plastic bags, taking four samples: (i) a silty surface (0-10 cm) sediment from the side of the Redon river (close to SP2 in Figure 1), 50 m from the lake, (ii) the soil (0-10 cm) from an artificial lawn used as a beach near the Redon mouth, (iii) a subsurface (depth 30-50 cm) silty-clay horizon from the eroded littoral path around the lake, and (iv) a sandy gravel littoral accumulation (0-10 cm) at the foot of a steep slope, close to Thonon-les-Bains.<br>We also used CTD temperature profiles, hyperspectral absorption (from AC-S; 81 channels between 400-730 nm), backscattering at 700 nm at 117° and chlorophyll-a fluorescence (from ECO Triplet BBFL2w), as well as Photosynthetically Active Radiation (PAR; between 400 to 700 nm; from Sea-Bird ECO PARs) collected by a WETLabs Thetis profiler.
Sampling strategy	Species identification and counting were performed in sedimentation chambers under an inverted microscope. Soil and sediment samples were dried at 35°C and sieved (2 mm).<br>The high-resolution (1-10 cm vertical resolution) profiles from Thetis were acquired at a 12-hr frequency during our study period, i.e., August-September 2021.
Data collection	Water samples were collected and analyzed by CIPEL, INRAE and University of Savoie Mont-Blanc. The Thetis data collection is conducted by the LéXPLORE consortium and is supported by the five involved partner institutions: University of Lausanne, EPFL, Eawag, University of Geneva, and INRAE-USMB (CARRTEL).
Timing and spatial scale	Water samples were collected on September 10, 2021 and September 13, 2021, a few days after the peak of the observed bloom. The high-resolution (1-10 cm vertical resolution) profiles from Thetis were acquired at a 12-hr frequency during our study period, i.e., August-September 2021.
Data exclusions	Less than 5% of the measurements by Thetis were identified as missing values, noise, or non-physical values due to instrumental artifacts (e.g., random electronic noise) or poor sample baselines, and were excluded.
Reproducibility	The results from remote sensing, in situ water sampling, and automated high-frequency profiles confirmed the peak of the observed bloom on 6 September, 2021.
Randomization	N/A
Blinding	N/A

Did the study involve field work? ☒ Yes ☐ No

Field work, collection and transport

Field conditions	The water samples were taken on calm and relatively warm days.
Location	Multiple locations and multiple depth in Lake Geneva (Switzerland/France)
Access & import/export	Chemical protocol was applied according to the sample specificity, and measurement performed on either the solid or the gas source, depending on the amount of carbon available in the sub-sample made in the lab. The detailed procedure is explained in the main manuscript.
Disturbance	N/A

Reporting for specific materials, systems and methods

We require information from authors about some types of materials, experimental systems and methods used in many studies. Here, indicate whether each material, system or method listed is relevant to your study. If you are not sure if a list item applies to your research, read the appropriate section before selecting a response.

Materials & experimental systems

n/a	Involved in the study	
☒	☐	Antibodies
☒	☐	Eukaryotic cell lines
☒	☐	Palaeontology and archaeology
☒	☐	Animals and other organisms
☒	☐	Clinical data
☒	☐	Dual use research of concern

Methods

n/a	Involved in the study	
☒	☐	ChIP-seq
☒	☐	Flow cytometry
☒	☐	MRI-based neuroimaging
